# The Effect of Orally Supplemented Melatonin on Larval Performance and Skeletal Deformities in Farmed Gilthead Seabream (*Sparus aurata*)

**DOI:** 10.3390/ijms21249597

**Published:** 2020-12-16

**Authors:** Kamel Mhalhel, Antonino Germanà, Francesco Abbate, Maria Cristina Guerrera, Maria Levanti, Rosaria Laurà, Giuseppe Montalbano

**Affiliations:** Zebrafish Neuromorphology Lab, Department of Veterinary Sciences, University of Messina, 98168 Messina, Italy; agermana@unime.it (A.G.); abbatef@unime.it (F.A.); mcguerrera@unime.it (M.C.G.); mblevanti@unime.it (M.L.); laurar@unime.it (R.L.)

**Keywords:** melatonin, opercular complex, bone deformity, growth, *Sparus aurata*, *PTHrP*, *mlc2*, *bglap*

## Abstract

The gilthead seabream larval rearing in continuous light is common in most Mediterranean hatcheries to stimulate larval length growth and increase food consumption. Several studies have shown that continuous light affects larval development and increases the prevalence of skeletal deformities. Melatonin is a crucial pineal neurohormone that displays daily secretion patterns, stimulates cell proliferation and embryonic development in Atlantic salmon and zebrafish, and improves osseointegration in mice and humans. However, no studies have examined the effects of orally supplemented melatonin on skeletal deformities in *Sparus aurata* larvae. We administered exogenous melatonin to gilthead seabream larvae via enriched rotifers and nauplii of Artemia. Exogenous melatonin induced bone deformities and stimulated parathyroid hormone-related protein-coding gene (*PTHrP*) mRNA expression. In addition to the melatonin-induced *PTHrP* high expression level, the recorded non coordinated function of skeletal muscle and bone during growth can be the fountainhead of bone deformities. Both myosin light chain 2 (*mlc2*) and bone gamma-carboxyglutamate protein-coding gene (*bglap*) expression levels were significantly affected by melatonin administration in an inverse dose–response manner during the exogenous melatonin administration. This is the first study to report the effect of inducing melatonin bone deformities on *Sparus aurata* larvae reared under ordinary hatchery conditions.

## 1. Introduction

The presence of morphological abnormalities in farmed gilthead seabream (*Sparus aurata*) is a major problem for current aquaculture as it entails significant economic losses [1,2,3]. Skeletal deformities are the most relevant deformities, and they include head and vertebral column anomalies. Opercular complex abnormalities are the most frequent skeletal anomalies in this species, with an incidence of 80% [2,3,4]. As the opercular complex’s function is the water ventilation and the gill’s protection, opercular deformities indirectly cause gill diseases by a lowered resistance to environmental stress [5]. Therefore, exposed gills can decrease respiratory efficiency, as well as reduce market value [2,5]. To improve the profitability of rearing gilthead seabream of commercial size, a rapid and early recognition of anomalies’ development is of prime importance for aquaculturists. A wide range of physical, chemical, and biological factors can cause skeletal deformities in farmed fish. Some authors have correlated the opercular deformities with a defect of inflation of the swim bladder [6], others have attributed it to a dietary deficiency of vitamins, amino acids [7]; or the rearing conditions (density, salinity, temperature, oxygenation, brightness) [8]. Additionally, according to some studies, heritability is central to the etiology of operculum complex anomalies [3]. Opercular complex deformities are induced during the embryonic and post-embryonic periods of life, long before osteological deformities are externally visible, and its development is still not well understood [9,10]. The larval rearing of gilthead seabream in continuous light is a common practice in the majority of Mediterranean hatcheries. Indeed, several studies have shown that continuous light accelerates the depletion of yolk sac reserves, stimulates larval length growth, and increases food consumption by around 40% [11]. However, several other studies have shown that continuous light affects larval development and increases the prevalence of skeletal deformities [12]. The circadian rhythm alternation has a major role in synchronizing daily behavioral processes in fish (locomotor activity, sedation, food intake, shoaling behavior), skin pigmentation, oxygen consumption, thermoregulation, and melatonin synthesis [13]. In fish, as in mammals, melatonin is the output signal of the circadian clock, a crucial hormone produced by the pineal gland that displays daily and seasonal patterns of secretion with a peak level during the dark-phase and basal level during the photo-phase [14]. The synthesis of melatonin also occurs in the retina. Retinal melatonin acts as a local neuromodulator within the eye, and it could be metabolized in situ, which prevents retinal melatonin from being released into the blood [14]. In addition, mRNA transcripts of melatonin synthesis enzymes have been reported in the digestive tracts of several teleosts with daily rhythms that adjust to the prevalent photoperiod [13,14,15,16]. The involvement of melatonin in larval development and growth has been demonstrated by several in vivo and in vitro studies [17,18,19,20,21,22] showing that melatonin stimulates cell proliferation and embryonic development in a dose-dependent manner in zebrafish [17,18]. Moreover, melatonin has several functions related to vertebral bone growth in Atlantic salmon [19], promotes osteoblastic differentiation in mice, human mesenchymal stem cells (MSC)/peripheral blood mononuclear cells and MC3T3-E1 cells by increased messenger RNA levels of osteogenic markers and improved osseointegration in mice [20,21,22]. In gilthead seabream larvae, melatonin production begins immediately after hatching to reach maximum levels between the 6th and 10th day after hatching [23]. At this time, levels of hormones involved in growth, metabolism and development, i.e., growth hormone and prolactin, are very low [24,25]. Additionally, it has been demonstrated that the number of growth hormone cells and growth hormone mRNA expression does not increase until 30 days post hatching [26]. When growth hormone and prolactin supply is still insufficient, melatonin may play a role in stimulation of cell proliferation and differentiation processes, as it has been postulated in zebrafish *Danio rerio* [18]. In light of all these bibliographic data, we thought that the inhibition of melatonin by continuous light, especially during the first days after hatching, increases the prevalence of skeletal deformities (especially operculum deformities) and alter larvae growth and development. To our knowledge, no study has been conducted on the effect of exogenous melatonin intake on the gilthead seabream skeletogenesis. Therefore, in the present study we evaluated the effect of an exogenous melatonin supplementation on skeletal deformities (especially the opercular complex abnormalities) and larval performance of gilthead seabream.

## 2. Results

### 2.1. Effect of Orally Supplemented Melatonin on Growth Rate

**Figure 1:**
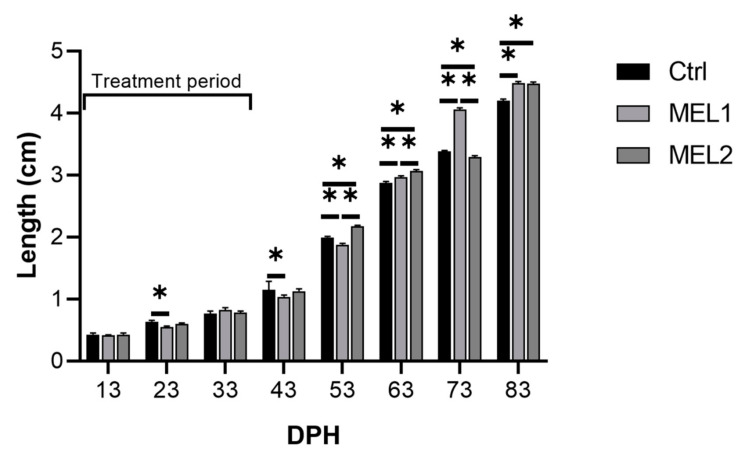
Total length variation in gilthead seabream larvae fed with rotifers (*Brachionus picatilis)* and Artemia (*Artemia salina*) enriched with two graded levels of melatonin (MEL1 and MEL2) (treatment period: from 3 to 38 days post hatching (DPH)). Data are expressed as mean ± SEM. Asterisks indicate significant differences between treatment and control groups (Mann–Whitney U test, *p* < 0.05).

**Figure 2:**
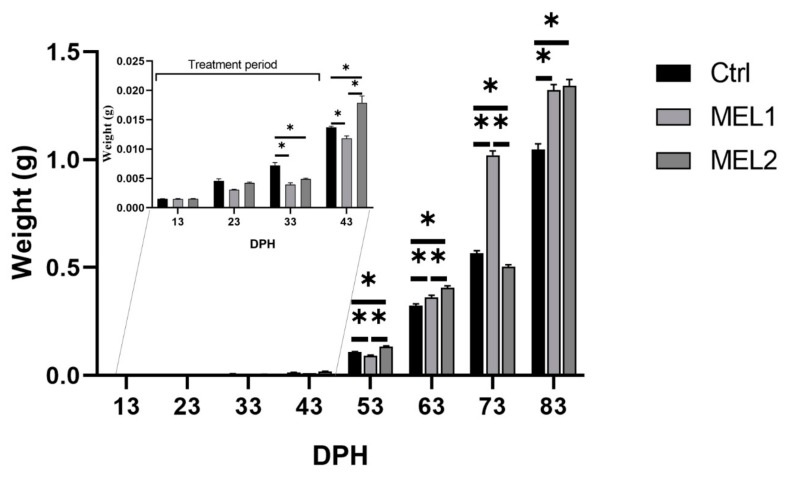
Weight variation in gilthead seabream larvae fed with (*Brachionus picatilis)* and Artemia (*Artemia salina*) enriched with two graded levels of melatonin (MEL1 and MEL2) (treatment period: from 3 to 38 DPH). Data are expressed as mean ± SEM. Asterisks indicate significant differences between treatment and control groups (Mann–Whitney U test, *p* < 0.05).

At 13 days post hatching (DPH), we did not record statistically significant differences between the three larvae groups for both length and weight (Figure 1 and Figure 2). At 23 DPH, the highest length was recorded in the Ctrl group (0.614 ± 0.0155 cm) which was statistically different only from MEL1 (Mann–Whitney U test, *p* < 0.05) (Figure 1). The growth in weight showed the same growth pattern in length and no statistically significant differences between all the experimental groups of larvae was recorded (Mann–Whitney U test, *p* < 0.05) (Figure 2). At 33 DPH, the Ctrl group showed the highest ponderal growth, while the MEL1 registered the lowest weight value. A significant difference between Ctrl and both treated groups for the weight was discerned (Mann–Whitney U test, *p* < 0.05) (Figure 1). However, those differences were absent for the growth in length (Figure 1). At 43 DPH, the length showed a statistically significant difference only between Ctrl and MEL1 (Mann–Whitney U test, *p* < 0.05). MEL2 displayed the highest growth in weight (0.0179 ± 0.001148 g), while MEL1 registered the lowest value. We noted statistically significant differences between the three groups of larvae for growth in weight (Mann–Whitney U test, *p* < 0.05) (Figure 2). At 53 DPH, the larvae of MEL2 group showed the highest length (2.17 ± 0.0163 cm), while MEL1 group larvae showed the lowest growth in length among the three groups of larvae (Figure 1). At this age, the weight distribution between the three groups corresponded well to the growth in length. The differences between the three groups for both weight and length were statistically significant (Mann–Whitney U test, *p* < 0.05) (Figure 1 and Figure 2). Ten days later, the larval growth in MEL1 exceeded that in Ctrl but was lower than that of MEL2 (Mann–Whitney U test, *p* < 0.05). At 73 DPH, the length in MEL1 continued to increase and reached 4.06 ± 0.0224 cm while in the Ctrl and MEL2 groups it did not exceed, respectively, 3.380 ± 0.0197 cm and 3.295 ± 0.197 cm (Figure 1). The length crest in MEL1 went with a weight crest of 1.021 ± 0.0196 g, which was 44.47%, and 50.66% higher, respectively, than that of the Ctrl group and MEL2 group (Mann–Whitney U test, *p* < 0.05). At the end of the experience, MEL1 and MEL2 larvae showed almost the same growth rate (4.48 cm/1.323 g–4.47 cm/1.342 g) and were statistically 6.40% longer and 22.38% heavier than that of the Ctrl group (Mann–Whitney U test, *p* < 0.05) (Figure 1 and Figure 2).

The length–weight relationship expressed by the equation W = aL^b^ was calculated in the three groups from the natural logarithmic equivalent log W = b log L + log a. The regression equation for Ctrl, MEL1, and MEL2 groups was, respectively, logW = 3.055 logL−4.383, log W = 1.087logL−1.18, logW = 0.954 logL−0.992. In both groups supplemented with melatonin, the b values (b_MEL1_ = 1.087, b_MEL2_ = 0.954) were inferior to three, which means that larvae fed with the different levels of melatonin gained less weight than the cube of its length, reflecting a negative allometric growth pattern and larvae were slimmer with increasing length. On the other hand, Ctrl group larvae showed a positive allometric growth pattern with a b value of 3.05, which means that while weight was still progressing, gains in length stopped.

### 2.2. Effect of Exogenous Melatonin on Insulin-Like Growth Factor 1 Concentration

**Figure 3:**
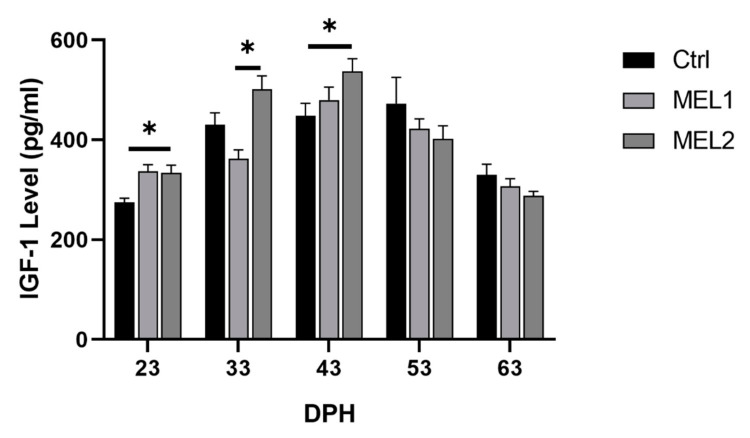
Insulin-like growth factor 1 (IGF-1) level of gilthead seabream larvae fed with rotifers (*Brachionus picatilis)* and Artemia (*Artemia salina*) enriched with two graded levels of melatonin (MEL1 and MEL2). Data are expressed as mean ± SD. Asterisks indicate significant differences between treatment and control groups (Mann–Whitney U test, *p* < 0.05).

The insulin-like growth factor 1 (IGF-1) concentration on gilthead seabream larvae fed with a graded level of MEL is shown in Figure 3. The bell-shaped histogram manifested peaks at 43 DPH, after which the IGF-1 rate dropped. At 23, 33, and 43 DPH, MEL2 larvae showed a higher concentration on IGF-1 than the Ctrl group. Still, that difference was statically significant only at 23 and 43 DPH (Mann–Whitney U test, *p* < 0.05). Additionally, MEL1 larvae showed a higher IGF-1 concentration than that of Ctrl group at 23 and 43 DPH but the differences were statically not significant. After the late metamorphosis (43 DPH), there were no statistically significant differences between the three experimental groups for the IGF-1 level (Mann–Whitney U test, *p* < 0.05).

### 2.3. Effects of Melatonin Oral Supplementation on the Ossification Pattern

**Figure 4:**
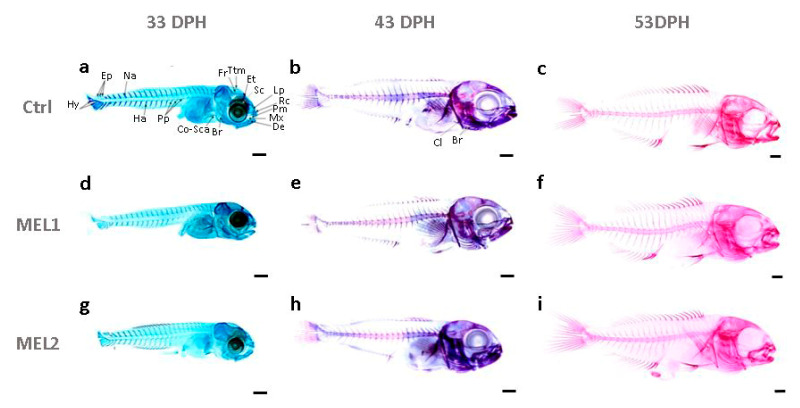
The ossification state in gilthead seabream larvae of Ctrl at 33 DPH (**a**), at 43 DPH (**b**), at 53 DPH(**c**); of MEL1 at 33 DPH (**d**), at 43 DPH (**e**), at 53 DPH (**f**); of MEL2 at 33 DPH (**g**), at 43 DPH (**h**), at 53 DPH (**i**). Double stained larvae showed alician blue-stained cartilaginous structures and alizarin red-stained bony structures. Br, branchiostegal rays; Cl, cleithrum; Co-Sc, coraco scapular cartilage; De, dentary; Ep, epural; Et, epiphysial tectum; Ha, hemal arches; Hy, hypural; Lp, lamina precerebralis; Mx, maxillary; Na, neural arches; Pm, premaxillary; Pp, parapophyse; Rc, rostral cartilage; Sc, sclerotic; Tmp, taenia marginalis posterior. Scale bar:0.5 mm.

The ossification state in cultured gilthead seabream larvae was studied using acid-free double staining. Figure 4 shows the chronology of the ossification in Ctrl, MEL1, and MEL2 larvae. Until 33 DPH, the double staining solution reveals only cartilaginous structures such as coraco scapular cartilage, branchiostegal rays, dentary, maxillary, premaxillary, rostral cartilage, lamina precerebralis, sclerotic, and epiphysial tectum in the cranial region (Figure 4a,d,g). On the vertebral column, we identified 23 neural arches and three epurals beside three parapophyses, 13 hemal arches, and five hypural cartilages (Figure 4a,d,g). At 43 DPH, all cartilaginous structures gained volume, and the ossification process was initiated in the dentary, maxillary, opercular complex on the head region (Figure 4b,e,h). A saltatory ossification process was initiated on the vertebral column on centra 1 to 4, 8 to 10, and 14 to 19 (Figure 4b,e,h). The peroration of ossification process occurred at 53 DPH when alizarin red staining had ascendancy over the larva (Figure 4c,f,i). Under the present experimental conditions, the same ossification patterns were observed between the three groups of larvae (Ctrl, MEL1, and MEL2), a continuous process (from 43 to 53 DPH) with the same ossification rate (Figure 4).

### 2.4. Opercular Complex Deformity: Gross Anatomy

**Figure 5:**
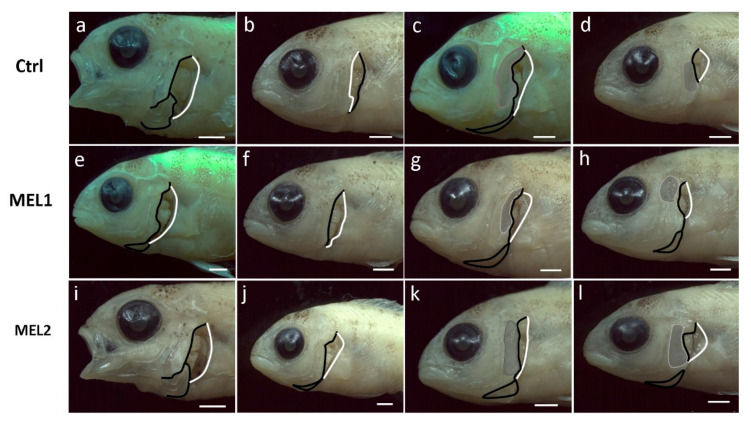
Some forms of opercular complex anomalies registered in Ctrl (**a**–**d**), in MEL1 (**e**–**h**), and in MEL2 (**i**–**l**) gilthead seabream larvae of 53 DPH. (**a**,**e**,**i**) Severe reduction in gills cover (opercle, subopercle, and interopercle); (**b**,**f**,**j**) severe reduction in gills cover (opercle and subopercle); (**c**,**g**,**k**) folded opercle upper corner and reduced subopercle and opercle lower corner; (**d**,**h**,**l**) folded gills cover leading to gills exposition. Black lines indicate the loose edge of the operculum and branchiestegal rays while white lines mark the limits of the normal pattern of the branchial chamber. Grey-shaded areas indicate folded bones. Scale bar: 1mm.

Since 53 DPH, larvae were completely ossified in Ctrl, MEL1, and MEL2, all forms of operculum complex anomalies at this stage were definitive. Figure 5 shows some forms of operculum complex deformities recorded in 53 DPH larvae from the three experimental groups. Opercular complex anomalies were registered as various gill cover irregularities and different degrees of gill chamber exposure. Those abnormalities can be the result of a reduction (Figure 5a,e,i), folding (Figure 5d,h,l), or both reduction and folding (Figure 5b,c,f,g,j,k) of one or more bones composing the opercular complex. The wide variability of malformation typologies arose in this study, and their severity did not allow the definition of accurate, specific patterns of the opercular complex deformities. Additionally, no type of abnormality was defined as group-specific.

**Figure 6:**
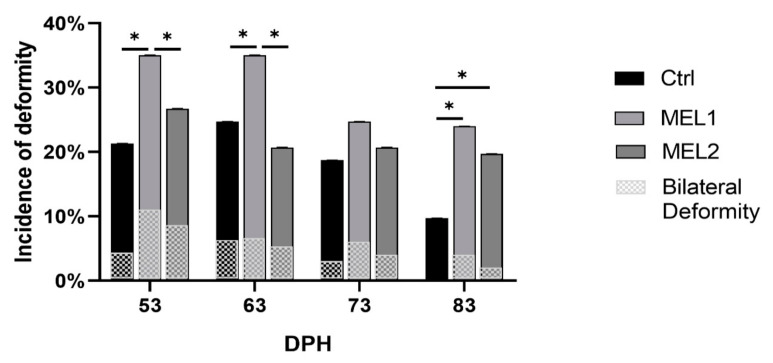
Incidence of opercular complex deformities on gilthead seabream larvae fed with rotifers (*Brachionus picatilis)* and Artemia (*Artemia salina*) enriched with two levels of melatonin (MEL1 and MEL2). Asterisks indicate significant differences between the three experimental groups of larvae (Chi-square test, *p* < 0.05).

The incidence of opercular complex deformities and the nature of this disorder as being unilateral or bilateral in the treated larvae of gilthead seabream are shown in Figure 6. The incidence of the opercular complex deformities in larvae fed the lower dose of MEL was significantly higher than those observed in the control group larvae (Chi-square test, *p* < 0.05) at 53, 63, and 83 DPH. Still, at 73 DPH, this difference was not statistically significant. Regarding the incidence of opercular complex deformities between MEL2 larvae and Ctrl group larvae, there was no significant statistical difference (Chi-square test, *p* < 0.05) at 53, 63, and 73 DPH, but there was at 83 DPH. In all groups, the anomalies were mostly unilateral (65.6 to 100% of cases) and (in some cases) were bilateral.

### 2.5. Opercular Complex Anomalies under Scanning Electron Microscope

**Figure 7:**
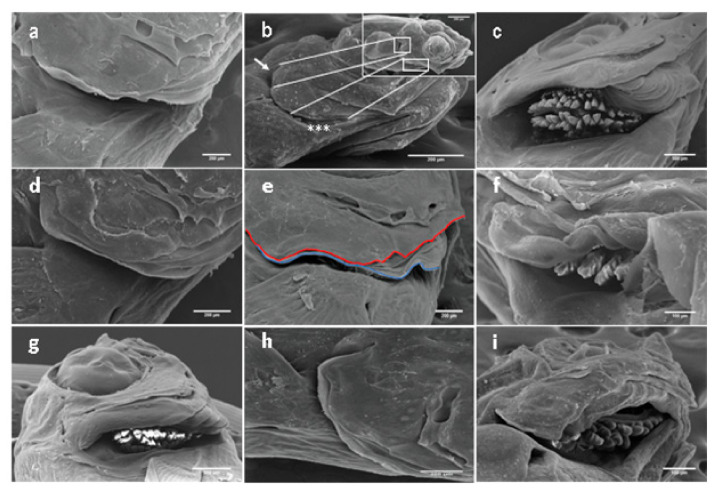
Gilthead seabream larvae aged 13-53 DPH. Different forms of abnormalities in the opercular complex. (**a**) Hypoplastic operculum, (**b**) reduced opercle (white arrows) and lack of interopercle (asterisks), (**c**) folded opercular bones toward the gills chamber leading to exposed gill arches, (**d**) outside folded operculum, (**e**) wave-like gill caver (red line) modulating a branchiostegal membrane (blue line), (**f**) spring-like gill caver with exposed gill arches, (**g**) combined shortened-folded operculum, (**h**) hyperplastic branchiostegal membrane, (**i**) folded branchiostegal rays in the gill chamber. Scale bars: 200 µm.

SEM observation provided a better perception and understanding of different forms of opercular complex deformity compared to stereomicroscope observation. The detected opercular complex anomalies involved the different opercular bones (opercle, subopercle, interopercle, and preopercle) and branchiostegal rays and membrane. The protective wall for the orobranchial chamber can be reduced in different levels, with references to a reduction or even lack of various bones of the operculum series (Figure 7a,b). Other types of anomalies are attributed to inside or outside folding of one or more opercular bones (Figure 7c,d) or a combined shortened-folded operculum (Figure 7g). Additionally, wave-like (Figure 7e) and spring-like (Figure 7f) gill cavers have been observed for the first time in some gilthead seabream larvae. We report, for the first time, the lack of apposition of the branchiostegal membrane to the epithelium at the terminal edge of the branchial cavity resulting from different degrees of hyperplasia (Figure 7h) and folded branchiostegal rays in the gill chamber (Figure 7i).

### 2.6. Skeletal Deformities

**Figure 8:**
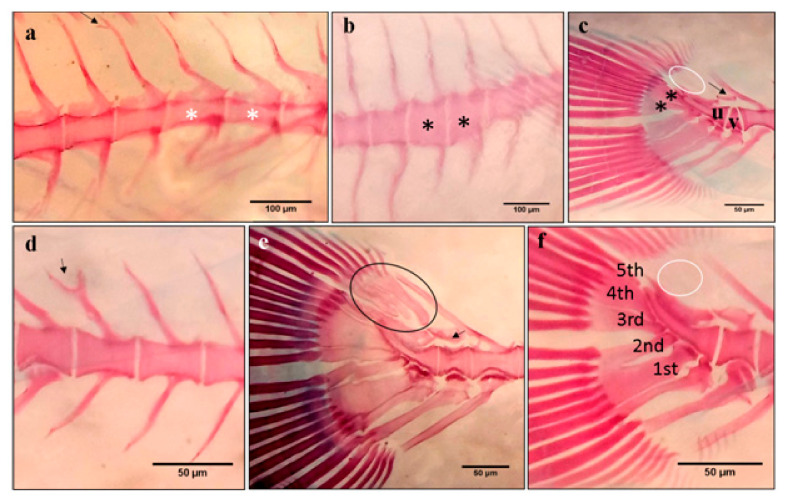
Representative forms of skeleton anomalies in gilthead seabream larvae from the different experimental groups at 53 DPH. (**a**) Rectangular slender vertebral body (white asterisks) and bifurcated neural spine (arrow), (**b**) cubic thick vertebral body (black asterisks), (**c**) last caudal vertebrae (v) preceding urostyle (u) is triangular shaped with detached neural spine (arrow) and the absence of the three epurals (white circle), lack of the fifth hypural and fusion of the third and fourth hypural (black asteriks), (**d**) bifurcated neural spine (black arrow), (**e**) detached neural spine (arrow). The circle indicates normal epural arrangement, (**f**) normal hypural arrangment (1st to 5th hypurals) and absence of the three epurals (white circle). Scale bars (**a**,**b**): 100 µm. Scale bars (**c**–**f**): 50 µm.

The early detection of skeletal deformities in cultured gilthead seabream larvae was studied using acid-free double staining (Figure 8).

The observation of double-stained larvae under a stereomicroscope revealed many typologies of bone deformities (Figure 8). Detected deformities affected meristic and threshold traits of the vertebral column (vertebrae, neural and hemal spin) and caudal fin complex. The vertebral column conserved its normal curvature (no lordose or kyphotiose cases were recorded), its meristics characteristics, and it was composed of 24 vertebrae in all larvae from the three experimental groups. However, several malformations affected vertebrae, and we retrieved a rectangular slender vertebral body (Figure 8a), cubic thick vertebral body (Figure 8b), and triangular-shaped vertebrae (Figure 8c). Neural and hemal spine also presented abnormalities such as: bifurcated neural spine (Figure 8a,d) and detached neural and hemal spine (Figure 8c,e). Skeletal deformities were also detected in the caudal portion, such as the absence of one or all of the three epurals (Figure 8c,f), and the lack, or fusion of hypural (Figure 8c). In many cases, we recorded multiple deformities in the same sample (Figure 8a).

The typology and incidence of skeletal abnormalities in gilthead seabream larvae fed with different levels of melatonin are shown in Table 1. Out of a total of 90 samples in each group (30 per each sampling point: 33, 43 and 53 DPH), 76 (84.4%), 83 (92.2%), and 87 (96.7%) from the Ctrl, MEL1, and MEL2 groups, respectively, showed at least one type of deformity, and we have recorded a significant statistical difference between the above-mentioned frequencies of Ctrl and MEL2 groups. Deformities affecting the caudal fin complex were the most common abnormalities encountered with 45.6% in Ctrl larvae, 62.2% in MEL1 group, and 77.8% in MEL2 larvae. The statistical difference between aforementioned frequencies was significant. The frequency of larvae with at least one vertebral anomaly was 23.3% in Ctrl and MEL2 groups and 28.9% in MEL1. No statistically significant difference was registered between the different groups. We have registered an incidence of hemal and neural spine deformities of 61.1%, 63.3%, and 55.6%, respectively, in Ctrl, MEL1, and MEL2 groups, and no statistically significant difference was registered between the different groups.

### 2.7. Effects of Exogenous Melatonin on Gene Expression

**Figure 9:**
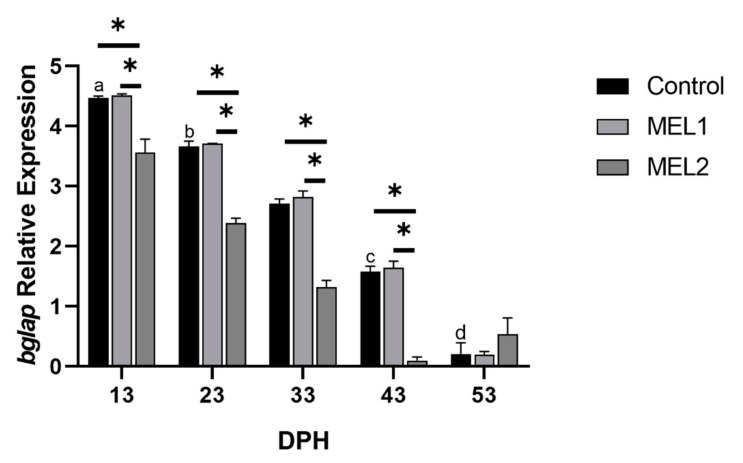
Gene expression of bone gamma-carboxyglutamate protein-coding gene (*bglap*) components in response to MEL administration in bone. Gene expression values are expressed as arbitrary units (a.u) for elongation factor 1-alpha (*ef1α*). Data are expressed as mean ± SD. Different letters indicate statistically significant differences throughout time within groups and asterisks indicate significant differences between groups for each treatment group (Welch test, *p* < 0.05).

Melatonin was administrated in two doses MEL1 and MEL2, for gilthead seabream larvae to study the response at the transcriptional level in osteogenesis and growth. Tissue-specific genes for bone—bone gamma-carboxyglutamate protein-coding gene (*bglap*), and parathyroid hormone-related protein-coding gene (*PTHrP*)—and for skeletal muscle—myosin light chain 2 (*mlc2*)—were analyzed. In most sampling points, the expression of both *bglap* and *PTHrP* was significantly affected by melatonin administration. Considerable variability in *bglap* transcript abundance was detected between treated groups, and between Ctrl and MEL2 as well, while the differences between Ctrl and MEL1 were statistically non-significant (Figure 9). Despite the significantly decreased expression of *bglap* over larval development, the expression pattern was conserved in all treatment groups of each sampling point, even after the end of melatonin administration (by starting weaning larvae onto dry pellets).

**Figure 10:**
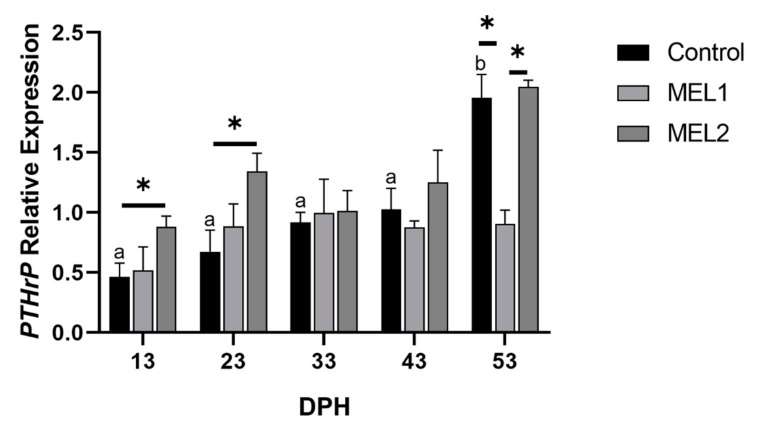
Gene expression of parathyroid hormone-related protein-coding gene (*PTHrP*) components in response to MEL administration in bone. Gene expression values are expressed as arbitrary units (a.u) for elongation factor 1-alpha (*ef1α*). Data are expressed as mean±SD. Different letters indicate statistically significant differences throughout time within groups and asterisks indicate significant differences between groups at each sampling point (Welch test, *p* < 0.05).

Unlike *bglap*, *PTHrP* showed an increasing expression over time. Furthermore, a dose–response relationship between melatonin concentration and *PTHrP* transcriptional level was recorded during the first 33 DPH (Figure 10). During the experimental period, when the melatonin was administrated via live feeds, the *PTHrP* transcriptional levels in MEL1 were comparable to those in Ctrl group. However, the transcriptional levels of the gene mentioned above in MEL2 samples showed a statistically significant difference compared to those in the Ctrl group at 13 and 23 DPH, which decreased by the end of melatonin treatment period (37 DPH) and ended by being compared to those of Ctrl samples.

**Figure 11:**
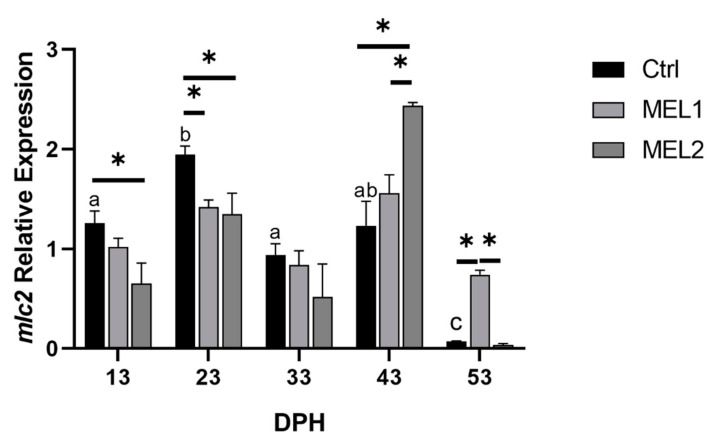
Gene expression of *mlc2* components in response to MEL administration in bone. Gene expression values are expressed as arbitrary units (a.u) for elongation factor 1-alpha (*ef1α*). Data are expressed as mean ± SD. Different letters indicate statistically significant differences throughout time within groups and asterisks indicate significant differences between groups for each treatment group (Welch test, *p* < 0.05).

As with *bglap* and *PTHrP*, *mlc2* showed considerable variability between control and treated groups. However, *mlc2* expression was significantly affected by melatonin administration in an inverse dose–response manner during the exogenous melatonin administration (Figure 11). At 43 DPH, an abrupt recovery of *mlc2* expression on treated groups temper the expression pattern in favor of a dose–response relationship with exogenous melatonin.

## 3. Discussion

Skeletal abnormalities and growth rates in gilthead seabream have been the subject of many research studies. Some authors have correlated the skeletal deformity with a defect of inflation of the swim bladder [6], others have attributed it to a dietary deficiency of vitamins [7], and fatty acids [27] as well as to the larval rearing systems [28], and rearing condition [8]. Additionally, the capacity of melatonin to induce embryonic development in zebrafish [17,18], and to control several functions related to bone growth in Atlantic salmon [19], was proved. In human adult mesenchymal stem cells/peripheral blood monocytes cocultures, MEL was able to induce osteoblastogenesis and suppress osteoclastogenesis by the effect of osteoblast-derived osteoblastic inhibitory lectin or by increasing osteoprotegerin and, receptor activator of nuclear factor-kB ligand ratios [20]. In mice osteoblast precursor cells (MC3T3-E1) as well, MEL induced osteoblast differentiation and bone formation by upregulating the gene expression of BMP2, BMP6, osteocalcin, and osteoprotegerin [21]. However, no study has evaluated exogenous melatonin effects on gilthead seabream skeletogenesis and growth during larval development. In the present study, the three groups of larvae (control group and two groups treated with different concentrations of melatonin) were placed under common hatchery conditions in continuous light to investigate the melatonin effect on skeletal abnormalities and growth rate in gilthead seabream. In teleosts, the melatonin synthesis reported on the pineal gland and gut has daily rhythms that adjust to the relevant photoperiod [14,15,16]. Additionally, the retinal melatonin acts as a local neuromodulator, and it is metabolized in situ. In light of this, we can gather that all the differences observed between the control and the two treatment groups of larvae maintained under continuous light were only due to the exogenous MEL incorporated into the commercial preparation of enrichment. The main finding from this study is the capacity of exogenous melatonin to affect gilthead seabream larval performance and the incidence of skeletal deformities. Thus, exogenous MEL affected normal skeletogenesis and caused bone deformities. Operculum complex and caudal fin complex were the most influenced structures. In gilthead seabream and many other species, operculum complex deformities are frequent [9,29,30,31,32]. The frequency of opercular anomalies on Ctrl groups (9.7 and 21.3%) was in the range of 6.3 to 43.2% as registered in previous studies conducted over the past two decades [9,30]. However, this represents almost a quarter of what was recorded in older studies [32]. Those differences may be attributed to the different rearing systems and conditions [9,28] and the acquisition of new knowledge in aquaculture. Additionally, the lower concentration of MEL affected the incidence of opercular complex deformities, while MEL2 did not. In fact, larvae fed with the lowest concentration showed the highest incidence of opercular complex deformities among the three groups. In agreement with Ortiz-Delgado et al. [9] and Berlado et al. [30], the deformities were mostly unilateral in all three groups (65 to 100% of cases). The wide variability of operculum deformity typologies, including the new forms registered in this study, hampered the establishment of specific patterns of deformities, like those defined by Berlado et al. [30]. In our study, the first sign of opercular complex alteration was detected on 13 DPH larvae, a long time before the ossification process took place, and earlier than that recorded by Galeotti et al. [29]. The exogenous melatonin concentrations increased caudal fin complex abnormalities in a dose-dependent manner. However, the control group had a frequency of 45.6% lower than 47.9% and 48.5% recorded in previous studies [33,34]. Additionally, the total frequency of individuals affected by at least one anomaly in the Ctrl group was 84.4%, which was lower than the 87% registered earlier [34]. The increased frequency of opercular complex, caudal fin complex, and total deformities might be a result of melatonin-induced osteoblasts accelerated differentiation already demonstrated on mice and human MSC/PBMC cells and MC3T3-E1 cells [20,21]. During teleost embryogenesis, cartilaginous structures are the first element of the skeleton to form, which becomes bone after mineralization [29,35,36]. The current study demonstrated that this pattern of development was valid for gilthead seabream larvae in which all skeletal elements remained cartilaginous until 43 DPH. Under the present experimental conditions, the same developmental patterns were observed between the three groups of larvae (Ctrl group, MEL1, and MEL2). The skeleton ontogenesis in the three groups of larvae revealed a priority to the onset of the elements which serve in feeding and respiration mechanisms (Maxillary, Meckel’s cartilage, cartilaginous branchial arches). These results agreed with those of earlier published studies [37,38]. Literature data on gilthead seabream meristic counts reported 24 vertebrae, 13 hemal arches, 23 neural arches, 4 pairs of parapophyses on the vertebral column region and 5 hypurals, 1 parahypural arch, 3 epurals on the caudal fin complex region beside all structures composing the cephalic skeleton [34,35,38]. In this study, the three experimental groups mostly conserved the meristics recorded in previous studies [34,35,38]. Exogenous MEL affected larval growth in length and weight in a dose-dependent manner. Indeed, MEL2 can ensure higher growth rates than those of the MEL1 and Ctrl groups. Additionally, both concentrations were in favor of a negative allometric growth pattern, which means that larvae were slimmer with increasing length. At 63 DPH, the Ctrl group had higher growth rates than those recorded in the previous studies [7,39]. The promising values recorded on the Ctrl group (high growth, the absence of lordotic kyphotic spine curve, and the lower opercular complex deformities), compared to the result already published for this species [9,30,33,35], indicated that the rearing conditions applied in the hatchery were optimal compared to those employed in the above-mentioned studies. IGF-1 has been associated with metabolism [40] and growth [41]. In some teleost species, tissue levels of its mRNA positively correlate with body growth rate [42]. Nevertheless, during this study, while the weight and total length continued to increase with age, the IGF-1 level increased until 43 DPH (shortly after the end of exogenous melatonin administration), after which it decreased. This variability registered during the first period (from 13 to 43 DPH) is in favor of an evident influence on IGF-1 production by the exogenous melatonin administration. In the present study, we investigated as well the exogenous melatonin-induced gene responses in skeletal muscle and bone tissue during the larval stage. The marker of muscle development and growth—myosin light chain 2 (*mlc2*)—one of the four light chains of the myosine, exerts its regulatory role binding calcium [43]. *bglap* is secreted by osteoblasts during bone formation and represents up to 1–2% of the total bone protein with a strong affinity for calcium [44]. *PTHrP* is a calcium regulatory factor in gilthead seabream, has a crucial function in several physiological and biochemical processes including cortisol production [45], tissue differentiation and proliferation [46], calcium mobilization from internal sources (bone and scales) and via calcium uptake from water and diet [47]. The lethal deletion of this gene on mice proves its crucial physiological functions [48]. The dose–response relationship between *PTHrP* expression level and melatonin concentration registered at least during the treatment period (MEL1 and MEL2) contradicts the negative correlation of those two parameters stipulated by Abbink et al. [49]. Indeed, another study on primary BALB/c mouse chondrocytes [50] supports our finding about melatonin’s capacity to upregulate *PTHrP* expression. Additionally, an association between abnormally developing bones and a high expression of *PTHrP* on *Sparus aurata* was already described [51]. In light of the aforesaid findings, we can assign the bone deformities registered in the treated groups to PTHrP expression product, which was induced by the orally supplemented melatonin. Myosin, a major component of striated muscle contributing to muscle contraction, is consisted of two heavy chains (MHCs) and four light chains (MLCs). Myosin light chain-2 is a sarcomeric protein which exists in white muscle of gilthead seabream as two isoforms *mlc2*a and *mlc2*b. Myosin light chain 2a predominates the early larval stages marking new fiber formation at the tissue level. In our study, the inverse dose-dependent relationship of *mlc2* expression levels and exogenous melatonin concentration was in accordance with the negative allometric growth pattern induced by melatonin, which means that larvae were slimmer with increasing length.

## 4. Materials and Methods

### 4.1. Larval Rearing

The experiment was conducted at the facilities of the hatchery AQUACULTURE TUNISIENNE at Sousse (Tunisia), according to the Guidelines on the Handling and Training of Laboratory Animals by the Universities Federation for Animal Welfare (UFAW). The experimental protocol was in accordance with the principles outlined in the declaration of Helsinki, and was approved by the local ethics committee (Identification code: 1400192; date of approval: 4 April 2017). Gilthead seabream larvae were raised under standard hatchery conditions in 2.5 m^3^ cylindroconical tanks (initial density of 120 larvae/L). Daily water renewal in the rearing tank was 3–15%/h with gentle and continuous aeration. The light intensity at water surface was 150 lux and the temperature was 20–21 °C. Rotifers *Brachionus picatilis* were cultured and enriched with green algae and added to tanks daily as an early live food from 3 to 25 DPH. *Artemia salina* nauplii were introduced from 26 to 38 DPH. One day before being fed to the larvae, rotifers and metanauplii were enriched with a commercial emulsion for enrichment Red Pepper (Bern Aqua, Olen, Belgium) with phytoproteins and highly unsaturated fatty acids. At 38 days, larvae were weaned onto dry pellets.

### 4.2. Experimental Design

The studies were carried out on 2400 gilthead seabream larvae, which were randomly divided after hatching into three groups of 800 larvae per group placed under ordinary hatchery conditions: a control group (Ctrl), and two melatonin-treated groups (MEL1) and (MEL2). The exogenous supply of melatonin was ensured via rotifers and nauplii of Artemia from 3 to 38 DPH. Rotifers and Artemia were enriched with 0.04 g/kg (MEL1) or 0.2 g/kg (MEL2) of neurohormone added in an ethanol solution to the commercial emulsion for enrichment. The choice of both concentrations was made considering the fact that no studies on melatonin administration have shown an LD50 (lethal dose for 50% of the subjects) [52], and considering as well the range of previously tested doses with positive responsiveness in other species of teleost [53,54,55]. The lipophilic nature of melatonin facilitates its solubility in this lipid emulsion. The experiment was performed in triplicate. Sampling was performed every ten days from 13 to 83 days post hatching (DPH) from each experimental group and larvae were sacrificed with an overdose of tricaine methane sulfonate (MS222); 1000–10,000 mg L^−1^.

### 4.3. Sampling

At each sampling point (from 13 to 83 DPH), 100 larvae per experimental group were collected for analyzing opercular complex abnormalities by stereomicroscope or scanning electron microscopy (SEM) for the smaller larvae (15 larvae of 13–53 DPH), the total length and weight measurement. Thirty larvae from those already investigated for operculum complex anomalies were double stained with alcian blue and alizarin red acid-free staining solution. For gene expression analyses, total RNA was extracted from pools of gilthead seabream larvae (5 to 20 individuals per tank depending on fish size).

### 4.4. Morphological Studies

At each sampling point, the study of operculum complex anomalies was performed using Leica M205C stereomicroscope (Leica, Milan, Italy) or scanning electron microscope Zeiss EVO LS 10 (Carl Zeiss NTS, Oberkochen, Germany) for the smaller larvae (larvae of 13-53 DPH: 15 larvae). Additionally, the total length was measured using a digital camera Leica IC80 HD (Leica, Milan, Italy) mounted on a stereo-microscope (Leica, Milan, Italy) and weight was determined with the analytical scale KERN 770 (KERN & Sohn, Balingen, Germany).

#### 4.4.1. Scanning Electron Microscopy

The samples were fixed in 2.5% glutaraldehyde in Sörensen phosphate buffer 0.1 M. After several rinsing steps in the same buffer, they were dehydrated in a graded alcohols series, critical-point dried in a Balzers CPD 030 (BAL-TEC AG, Balzers, Liechtenstein), sputter coated with 3 nm gold in a SCD 050 sample coater (BAL-TEC AG, Balzers, Liechtenstein) and examined under a Zeiss EVO LS 10 (Carl Zeiss NTS, Oberkochen, Germany) as described by Abbate et al. [56].

#### 4.4.2. Acid-Free Double Staining

The double staining procedure was performed according to an adjusted protocol of Walker and Kimmel [57]. Acid-free double staining solution was made in two parts. The first part for cartilage staining was done by adding together 0.2% alcian blue 8 GX (C.I. 58,005 from Sigma, St. Louis, MO, USA) in 70% ethanol, and 100 mM MgCl2 (for mucosubstances differentiation by competition with alician blue for the negative charges of acidic mucopolysaccharides). The second part for bone staining was 0.5% alizarin red S (C.I. 74,240 from Sigma, St. Louis, MO, USA). The final acid-free staining solution contained 10 mL of the first part and 100 µL of the second. Larvae of 23, 33, 43 and 53 DPH were fixed for 2 h in 4% paraphormaldehyde in phosphate buffered saline (PBS). After washing, larvae were dehydrated with 1 mL ethanol 50%, at room temperature for 10 min. After removing the ethanol, specimens were transferred directly to the acid-free double stain solution, and rocked at room temperature overnight to incorporate the stain adequately. Next, pigmentation was removed at room temperature for 20 min with a bleach solution made of 1.5% H_2_o_2_ and 1% KOH. Clearing was achieved by transferring specimens in 20% glycerol and 0.25% KOH solution overnight after which the solution was replaced with 50% glycerol and 0.25% KOH solution (overnight). Larvae were viewed and photographed with M205C stereomicroscope (Leica, Milan, Italy) in the same solution.

### 4.5. IGF-1 Quantification

IGF-1 quantification was performed using tissue homogenates (25% *w*/*v*) made from 40–55 larvae in each experimental group. The tissue homogenates were prepared in 0.05 mol/L phosphate buffered saline, pH 7.4 with the use of a knife homogenizer (Polytron) at 4 °C. Homogenates were centrifuged at 16,000× g for 20 min at 4 °C. Supernatant (tissue extract) was used for assays.

The quantification of IGF-1 in larvae was performed using Fish IGF-1 ELISA kit (catalog Num. CSB-E12122Fh, Cusabio Biotech, Wuhan, China) according to the manufacturer’s instructions. The assay employs the competitive inhibition enzyme immunoassay technique. The microtiter plate provided in the kit was pre-coated with goat-anti-rabbit antibody. Standards and samples were added to the appropriate microtiter plate wells with an antibody specific for IGF-1- and horseradish peroxidase (HRP)-conjugated IGF-1. The competitive inhibition reaction is launched between HRP-labeled IGF-1 and unlabeled IGF-1 with the antibody. A substrate solution was added to the wells and the colorimetric reaction developed by negative correlation with the amount of IGF-1 in the sample. The color development was stopped and the intensity of the color was measured.

### 4.6. Gene Expression Analyses

Gene expression analyses were carried out in compliance with the minimum information for publication of quantitative real-time pcr experiments, MIQE guidelines [58]. Total RNA was extracted from pools of gilthead seabream larvae (5 to 20 individuals per sample time and tank depending on fish size), in which head regions were taken off and muscle/bone tissues were separated, using the TRIzol reagent (Invitrogen, Carlsbad, CA, USA); then, 2 µg of total RNA was reverse transcribed into cDNA using the High-Capacity cDNA Archive Kit (Applied Biosystems, Foster City, CA, USA). The mRNA levels of bone-specific genes, bone gamma-carboxyglutamate protein-coding gene (*bglap*), parathyroid hormone-related protein-coding gene (*PTHrP*) and skeletal muscle specific gene, myosin light chain 2 (*mlc2*), were analyzed by qPCR using a SYBR^®^ Premix DimerEraser™ (Perfect Real Time) (Cat. #RR091A, TAKARA BIO INC, Otsu, Shinga, Japan). The primers were designed based on the published gilthead seabream mRNA sequences for the genes analyzed. Table 2 lists the GenBank accession numbers and primer sequences. Reactions were performed in triplicate using a 7500 PCR real-time system (Applied Biosystems). The results were calculated using the 2-ΔΔCt algorithm against elongation factor 1 alpha (*ef1α*) and expressed as the n-fold difference compared to an arbitrary calibrator, chosen as a higher value than ΔΔCts. Cycling parameters were as follows: 95 °C for 10 min, (95 °C for 15 s, Ta for 30 s, 72 °C for 40 s) (40 cycles), 95 °C for 15 s at the end of the amplification phase.

### 4.7. Statistics

Statistical analyses of all parameters were performed in IBM SPSS Statistics for Windows version 22, (IBM Corp, Armonk, NY, USA). Normality was analyzed using the Shapiro–Wilk test and homogeneity of variance using Levene’s test. Statistical significance was assessed by Chi-Square test for incidence of bone deformities, by Mann–Whitney U Test for growth parameters and Welch test for gene expression analysis. Values were considered statistically significant when *p* < 0.05. Data calculated for each group were expressed as mean± Standard error of the mean (SEM) or mean± standard deviation (SD).

## 5. Conclusions

Our data showed that the different MEL concentrations, and predominately the high one, affected the normal process of skeletogenesis, and the growth patterns on gilthead seabream larvae. MEL increased the frequency of skeletal abnormalities, especially those of the operculum complex, for which we have recorded new typologies. The first signs of the operculum complex deformity were recorded before any sign of mineralization, proving that the abnormality occurs during the onset of the operculum complex bone series. Caudal fin complex was also sensitive to exogenous melatonin administration in a dose-dependent manner. In light of our results and bibliographic data, we hypothesize that skeletal deformities detected in experimental groups’ larvae can be induced by the increased *PTHrP* expression level, which is upregulated by exogenous melatonin administration, or by a loss of co-ordination between skeletal muscle and bone function. Future studies should extend and unmask the melatonin-regulated pathways in gilthead seabream bone.

## Figures and Tables

**Table 1:** The incidence of bone anomalies on gilthead seabream larvae of the three experimental groups.

	Ctrl	Mel1	MEL2
Total abnormalities	84.4%a	92.2%ab	96.7%b
Vertebral abnormalities	23.3%	28.9%	23.3%
Hemal and neural spine abnormalities	61.1%	63.3%	55.6%
Caudal portion abnormalities	45.6%a	62.2%b	77.8%c

Different letters within the same row show statistically significant differences (Chi-square test, *p* < 0.05).

**Table 2:** Primers used for real-time quantitative PCR. F, Forward primer, R, reverse primer; Tm, annealing temperature.

Gene	Primer Sequences (5′-3′)	Tm°C	Accession Number
*bglap*	F: AGT GAC AAC CCT GCT GAT GA	58	AF289506
	R: TCC CTC AGT GTC CAT CAT GT		
*PTHrP*	F: CCC AGA GCC AAA CAT TCA GT	58	AF197904
	R: CGG CCT AAC CTC ACC TTT TT		
*mlc2*	F: TGG CAT CAT CAG CAA GGA	54	AF150904
	R: TTG AAA GCG CTC ACG ATG		
*ef1α*	F: CTTCAACGCTCAGGTCATCA	58	AF184170
	R: GTGGGTGCAGTTTGACAATG		
